# Context-dependent autoprocessing of human immunodeficiency virus type 1 protease precursors

**DOI:** 10.1371/journal.pone.0191372

**Published:** 2018-01-16

**Authors:** ChihFeng Tien, Liangqun Huang, Susan M. Watanabe, Jordan T. Speidel, Carol A. Carter, Chaoping Chen

**Affiliations:** 1 Department of Biochemistry and Molecular Biology, Colorado State University, Fort Collins, Colorado, United States of America; 2 Department of Molecular Genetics and Microbiology, Stony Brook University, Stony Brook, New York, United States of America; "INSERM", FRANCE

## Abstract

HIV-1 protease autoprocessing is responsible for liberation of free mature protease (PR) from the Gag-Pol polyprotein precursor. A cell-based model system was previously developed to examine the autoprocessing mechanism of fusion precursors carrying the p6*-PR miniprecursor sandwiched between various proteins or epitopes. We here report that precursor autoprocessing is context-dependent as its activity and outcomes can be modulated by sequences upstream of p6*-PR. This was exemplified by the 26aa maltose binding protein (MBP) signal peptide (SigP) when placed at the N-terminus of a fusion precursor. The mature PRs released from SigP-carrying precursors are resistant to self-degradation whereas those released from SigP-lacking fusion precursors are prone to self-degradation. A H69D mutation in PR abolished autoprocessing of SigP-containing fusion precursors whereas it only partially suppressed autoprocessing of fusion precursors lacking SigP. An autoprocessing deficient GFP fusion precursor with SigP exhibited a subcellular distribution pattern distinct from the one without it in transfected HeLa cells. Furthermore, a SigP fusion precursor carrying a substitution at the P1 position released the mature PR and PR-containing fragments that were different from those released from the precursor carrying the same mutation but lacking SigP. We also examined autoprocessing outcomes in viral particles produced by a NL4-3 derived proviral construct and demonstrated the existence of several PR-containing fragments along with the mature PR. Some of these resembled the SigP precursor autoprocessing outcomes. This finding of context-dependent modulation reveals the complexity of precursor autoprocessing regulation that most likely accompanies sequence variation imposed by the evolution of the upstream Gag moiety.

## Introduction

The Human Immunodeficiency Virus type 1 (HIV-1) protease (PR) is one of the three indispensable enzymes encoded by the viral genome with the other two being reverse transcriptase (RT) and integrase (IN). In the HIV-infected cell, these enzymes are initially synthesized as part of the Gag-Pol polyprotein precursor which shares the same N-terminus with the Gag structural precursor polyprotein. Within the Gag-Pol precursor, PR is flanked by an upstream peptide sequence and by the downstream RT. The upstream peptide is named transframe region (TFR) or p6* [1, 2] as its coding sequence overlaps with the p6 region of the Gag reading frame. During the late stage of virus replication, Gag and Gag-Pol co-assemble into virus particles that subsequently bud off from the infected cell. Upon or shortly after virion release, the Gag-Pol polyprotein undergoes autoproteolysis and liberates free mature PR–a process generally referred to as PR autoprocessing.

Two cleavage reactions are necessary to release mature PR from its Gag-Pol polyprotein precursor: one at the N-terminus and the other at the C-terminus. Blocking the C-terminal cleavage leads to production of a PR-RT fusion enzyme that still supports productive viral replication [3]. In contrast, blocking the N-terminal cleavage leads to production of p6*-PR that exhibits limited proteolytic activity but is incapable of producing infectious virions [4]. Removal of the p6* region is concurrent with the appearance of mature PR activity *in vitro* and p6* deletion also enhances *in vitro* PR autoprocessing [1, 5]. Based on several lines of study, the cleavage between p6* and PR is a critical step for liberation of fully active mature PR [6–9] and the p6*-PR is thus defined as a miniprecursor.

**Fig 1:**
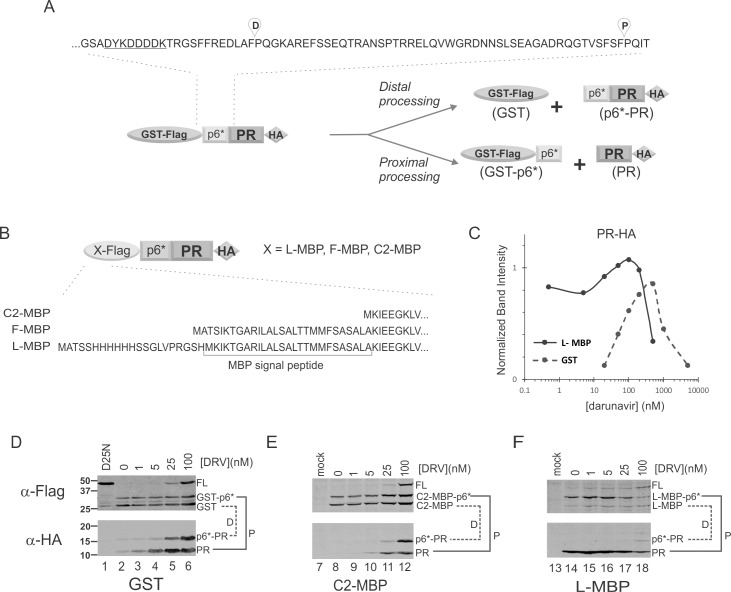
MBP fusions modulate autoprocessing products. **(A)** A schematic diagram illustrating prototypic GST fusion precursor distal and proximal processing sites, and the corresponding products. The Flag epitope sequence is underlined. (**B)** Domain organization and N-terminal sequences of MBP fusion precursors. (**C)** Quantification of the mature PRs released from GST- (dashed line) or L-MBP- (solid line) precursors in transfected HEK293T cells. The mature PR was detected with mouse anti-HA antibody and normalized to GAPDH signal to reflect its steady-state amount. The graph is representative of five independent experiments. (**D-F)** Autoprocessing of various fusion precursors at low DRV concentrations (<100nM). The corresponding proximal (P) and distal (D) processing products are denoted and linked by the solid and dashed lines, respectively.

HIV-1 protease autoprocessing is an intriguing process in that the Gag-Pol polyprotein precursor serves as both the substrate and the enzyme prior to liberation of the mature PR. We recently developed a model system to study the autoprocessing mechanism by expressing fusion precursors in transfected mammalian cells [7–9]. A typical fusion precursor consists of the p6*-PR miniprecursor (derived from the NL4-3 strain) sandwiched between GST and a small epitope peptide such as HA derived from the Influenza virus hemagglutinin protein (Fig 1A). This model system allows us to directly examine precursor-mediated liberation of mature PR from the fusion precursors. With this model system, we have reported that precursor autoprocessing is more resistant than mature PR to suppression by known PR inhibitors [7, 8], which is consistent with previous observations made with purified recombinant miniprecursor [10–12] or *in vitro* translated Gag-Pol polyprotein precursor [13]. Thus, this model system provides an easy and simple tool for the study of the precursor autoprocessing mechanism.

We previously tested several other tags in the place of GST and found that all the resulting fusion precursors are autoprocessing competent [8]. This raised a fundamental question: is p6*-PR miniprecursor autoprocessing autonomous and independent of the flanking sequences? To address this question, we examined fusion precursors carrying slightly different versions of maltose binding protein (MBP) at the N-terminus. Strikingly, our study demonstrated that precursor autoprocessing outcomes are context-dependent. The mature PRs released from different fusion tags demonstrated distinct self-degradation properties and H69D point mutation displayed different effects on precursor autoprocessing activity when fused to different tags. Furthermore, the MBP derived 22 aa signal peptide (SigP) modulated precursor autoprocessing in a manner similar to a NL4-3 derived proviral construct. Therefore, our study provides evidence suggesting that precursor autoprocessing is a context-dependent process and can be modulated by sequences beyond the p6*-PR region to release autoprocessing products with distinct enzymatic properties.

## Materials and methods

### DNA mutagenesis

All the plasmids used in this study were constructed by the standard PCR-mediated mutagenesis and cloning procedures. The parental GST and L-MBP fusion plasmids were previously described [7, 14, 15] and each engineered mutation made for this study was verified by sequencing analysis (see S1 File for details). A standard agreement is followed for any material transfer.

### Cell culture and transfection

HEK 293T cells were purchased from ATCC (Manassas, VA) and maintained in DMEM medium containing 10% fetal bovine serum, 100 units/ml of penicillin G sodium salt and 100 μg/ml of streptomycin sulfate. Transfection of HEK293T cells by calcium phosphate was described previously [7, 15, 16]. In brief, HEK 293T cells were seeded in a 12-well the day before transfection to achieve 30–40% confluence at the time of transfection. Chloroquine was added into each well to a final concentration of 25μM. DNA-Calcium mixture was first made by mixing a total of 0.5 μg plasmid DNA in 65.7 μl H2O with 9.3 μl of 2 M CaCl2. Then 75 μl of 2 x HBS (50 mM Hepes, pH7.04~7.05, 10 mM KCl, 12 mM Dextrose, 280 mM NaCl, and 1.5 mM Na_2_HPO_4_) was added slowly to the DNA-Calcium mixture with gentle vortex. The resulting mixture was then added to each well dropwise. At 7–11 hours post transfection, the culture medium was replaced with fresh chloroquine-free medium with or without PR inhibitors at the indicated concentrations. At about ~30 h post-transfection, cells were gently rinsed with 1x PBS once, and lysed in situ by adding 100 μl lysis buffer A (Tris-HCl, pH8.0, 150mM NaCl, 1% sodium deoxycholate, and 1% Triton X-100) with protease inhibitor cocktail. The cell lysates were collected and subjected to a brief centrifugation (10,000 x g for 2 min) to remove host chromosomes. The resulting post-nuclear supernatants were directly analyzed by western blot or stored at -20°C.

### Immunoprecipitation and N-terminal sequencing of PR fragments

HEK293T cells transfected with L-MBP-p6*-F56C-PR^L63P^-HA encoding plasmid grown in 3 x T175 flasks were collected by trypsin-EDTA treatment followed by a brief centrifugation (1000 x g for 5 min). The cell pellet was then lysed in 9 ml of 1x PBSX (1% Triton X-100 made in 1x PBS) containing 1 μM indinavir to prevent autoproteolysis. The cell lysate was first incubated with 120 μL of amylose beads (BioLabs, cat# E8021) at 4°C overnight to absorb MBP-containing fragments including the full length unprocessed precursor. The remaining supernatant was then mixed with 150 μL anti-HA slurry (Sigma, cat# A2095). After overnight incubation at 4°C, the beads were washed three times with 1x PBS buffer and the associated proteins were recovered by boiling the beads in 250 μL 1x SDS loading buffer. The immunoprecipitated proteins were resolved in 13% SDS-PAGE and transferred onto a PVDF membrane that was subsequently stained with Ponceau S for 5 min followed by two rinses with Nanopure water and air dried. The stained bands were excised out from the membrane and loaded onto a 494 Procise Protein Sequencer/140C Analyzer from Applied Biosystems for N-terminal sequencing by Edman degradation.

### Microscopic analysis of precursor subcellular distribution

HeLa cells grown on poly-L-lysine coated cover slips were transfected with the eGFP fusion precursor encoding plasmids with X-treme Gene Transfection Reagent (Roche). After 24h incubation, cells were fixed in 4% formaldehyde, nuclei were stained with Hoechst and preserved with diamond antifade mountant (Invitrogen). Images were captured on an inverted fluorescence/differential-interference contrast (dic) Zeiss Axiovert 200M deconvolving fluorescence microscope operated by AxioVision Version 4.5 (Zeiss) software and deconvolved by using the constrained iterative method (AxioVision).

### Viral particle production and collection

HEK293T cells grown in 6-well plates were transfected as previously described [7, 15, 16]. The culture medium was replaced at 7–11 h post-transfection and the HIV-1 protease inhibitor indinavir was added to the indicated concentrations. At ~50 h post-transfection, the culture supernatants containing viral particles were clarified by centrifugation at 20,800 × g for 2 minutes and the particles were then pelleted at 20,800 × g for 2 h through a 20% (wt/vol in phosphate-buffered saline (PBS)) sucrose cushion. The pelleted particles were resuspended in 25 ul of 1.5x SDS loading buffer and subjected to SDS-PAGE analysis followed by western blotting.

### SDS-PAGE and western blotting

Approximately equal volumes of the post-nuclear lysates were resolved by SDS-PAGE followed by protein transfer to a PVDF membrane. Primary antibodies used in this study include rabbit anti-GST (Sigma, cat# G7781), mouse monoclonal anti-HA (Sigma, cat# H9658), anti-flag (Sigma, cat# F1804), anti-GAPDH (Millipore, cat# MAB374), mouse monoclonal anti-p24 [17, 18], and rabbit anti-PR (NIH AIDS Reagent Program, cat# 4105). Secondary antibodies included IR800 fluorescence labeled goat anti-rabbit (Rockland, cat#611-132-003) and IR800 goat anti-mouse (Rockland, cat# 610-132-121). The blots were visualized with an Odyssey infrared dual laser scanning unit (LI-COR Biotechnology, Lincoln, Nebraska). To reduce background noise in some blots, the primary antibody was first absorbed against cell lysates made from untransfected 293T cells that were resolved by SDS-PAGE and transferred onto a PVDF membrane. Then the pre-absorbed supernatant was used to probe the blots containing the test lysates.

## Results

Our prototypic fusion precursor consists of the p6*-PR miniprecursor sandwiched between GST and HA tags. The NL4-3 derived p6*-PR has two autoproteolysis sites (Fig 1A). One is between p6* and PR, designated as the proximal (P) site, which is also equivalent to the N-terminal processing site essential for liberation of mature PR. The other one is located at the N-terminal region of p6*, defined as the distal (D) site in this study. Precursor autoprocessing at the proximal site produces GST-p6* and PR (with PQIT at N-terminus); whereas precursor autoprocessing at the distal site released GST and p6*-PR (with PQGK at N-terminus). With GST fusion, these two sites are equally processed as indicated by detection of approximately equal amounts of GST (from distal processing) and GST-p6* (from proximal processing) products (Fig 1D α-Flag panel as an example). On the other hand, p6*-PR and PR are normally undetectable due to rapid self-degradation in the absence of any PR inhibitor [19, 20]. These PR-containing products become detectable when self-degradation is suppressed by PR inhibitor treatment (Fig 1D α-HA panel).

### Mature PR released from L-MBP fusion precursor is self-degradation resistant

To investigate whether p6*-PR autoprocessing could be affected by the upstream flanking sequences, we tested maltose binding protein (MBP) as a fusion tag in three slightly different versions (Fig 1B). The F-MBP has the full-length coding sequence derived from *E*. *coli*, including the signal peptide (SigP) responsible for MBP export into the periplasm. The L-MBP has a 6xHis tag fused to the N-terminus of F-MBP through a short linker. The C2-MBP lacks the SigP and is widely used to solubilize fusion proteins in *E*. *coli* and mammalian cells [21–24].

The C2-MBP precursor displayed autoprocessing phenotypes in response to PR inhibitor treatment like the GST precursor (Fig 1D and 1E). Both were autoprocessing competent and processed the P and D sites at approximately equal rates. The mature PRs released from these two fusion precursors exhibited a characteristic bell-shaped detection profile when treated with increasing darunavir (DRV), a potent protease inhibitor (Fig 1C, dashed line). The released mature PR is normally undetectable due to self-degradation [19, 20] in the absence of a PR inhibitor. Low DRV concentrations (<500 nM) suppressed PR self-degradation without affecting precursor autoprocessing, resulting in increased detection of the mature PR. As the DRV concentration is increased further, precursor autoprocessing is inhibited leading to less production of the mature PR. Comparable results were also obtained with saquinavir (SQV), another PR inhibitor (data not shown).

The L-MBP precursor exhibited different autoprocessing phenotypes compared to the C2-MBP and GST precursors. The mature PR released from L-MBP precursor was readily detectable in the absence of PR inhibitor and remained so at low DRV concentrations (<100nM), suggesting that it was not rapidly self-degraded as those released from GST and C2-MBP precursors (Fig 1F, α-HA panel). N-terminal sequencing analysis of the mature PR made by the L-MBP precursor verified the PQITL N-terminus as a typical mature PR. Furthermore, F-MBP precursors also released mature PRs that were resistant to self-degradation (data not shown). Therefore, the mature PRs released from L-MBP and F-MBP precursors displayed a distinct self-degradation property unlike those released from GST and C-MBP precursors (Fig 1C). Our data suggest that p6*-PR precursor autoprocessing outcomes could be influenced by different contexts, *i*.*e*., fusion tags, such that the L-MBP- and the GST- (or C2-MBP-) precursors may have different intrinsic proteolytic activities and therefore release mature PRs with different properties. It should be noted that not all precursors undergo productive autoprocessing with release of anticipated autoprocessing products; a portion of them may directly undergo self-degradation leaving no trace of any defined products. We focused our quantification analysis on the released mature PRs (Fig 1C), which demonstrated different outcomes illustrating the context-dependent characteristic of precursor autoprocessing.

### L-MBP fusion abolishes H69D autoprocessing

**Fig 2:**
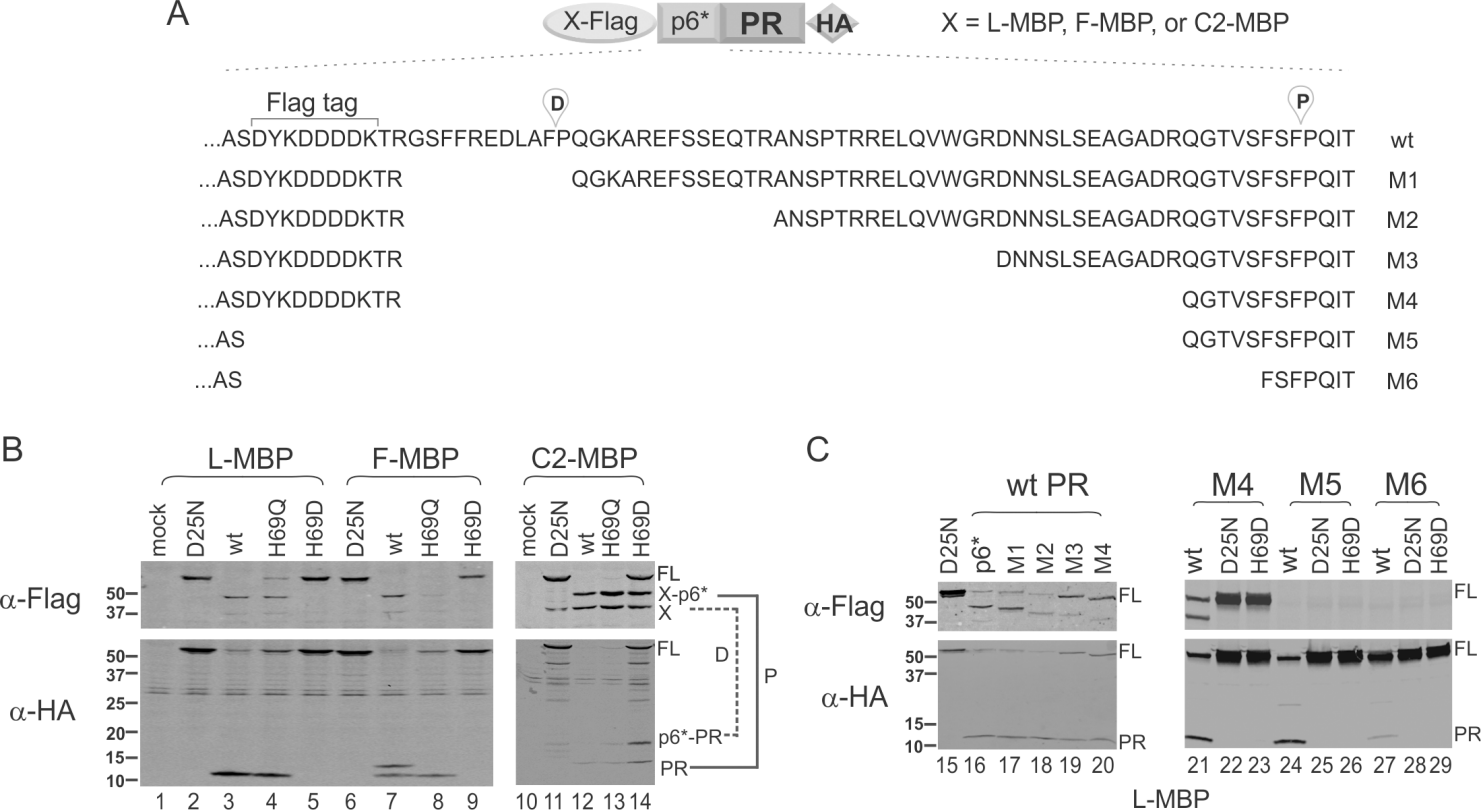
H69D mutation abolishes autoprocessing in the context of L-MBP/F-MBP fusion independent of p6* sequences. **(A)** Schematic illustration of the tested constructs. (**B)** Autoprocessing of H69 mutants in the context of MBP fusions. Approximately equal amounts of lysate were probed with mouse anti-Flag and anti-HA antibodies in parallel. The corresponding proximal (P) and distal (D) processing products are denoted and connected by the solid and dashed lines, respectively. (**C)** Autoprocessing of L-MBP fusion precursors carrying p6* truncations.

We previously reported that H69D mutation completely suppresses PR autoprocessing in the context of a NL4-3 proviral construct but only partially inhibits precursor autoprocessing in the context of GST fusion [7, 8, 16]. We speculated that this context-dependent discrepancy was indicative of a modulating determinant(s) associated with the NL4-3 proviral sequence but absent in the GST fusion precursor. In the light of different autoprocessing outcomes mediated by L-MBP vs C2-MBP fusion, we examined the possibility that H69D autoprocessing could also be differentially affected by the sequence upstream of p6*. We also included H69Q as another control as it demonstrated wild type autoprocessing activity in our previous reports [7, 8, 16]. As shown in Fig 2B, with C2-MBP fusion H69D autoprocessing was partially inhibited as indicated by detection of autoprocessing products plus the full length precursor (Fig 2B, *lane 14*), which was similar to the outcome of GST fusion [7, 8, 16]. In contrast, with L-MBP fusion H69D completely suppressed precursor autoprocessing as only the full-length precursor was detected and was comparable to the D25N control (Fig 2B, lane 5). These results indicate that H69D autoprocessing is differentially affected by the L-MBP vs C2-MBP tag. Therefore, both proviral context and L-MBP fusion abolish H69D autoprocessing although the underlying mechanism remains to be defined.

We next asked whether the p6* peptide has a role in modulating H69D autoprocessing activity by testing L-MBP precursors with various p6* truncations. Our data revealed that the mature PRs released from these L-MBP precursors remained readily detectable (Fig 2C, lanes 16–20) and H69D mutation also fully abolished autoprocessing activity of these precursors (Fig 2C, lanes 23, 26, 29). The M5 and M6 constructs do not have the Flag tag (Fig 2A) and thus are only detectable by HA antibody (Fig 2C). The M6 precursor has only the last 3 residues of p6* and was barely active at autoprocessing (Fig 2C, lane 27), likely due to disruption of the substrate sequence at the proximal site. In any case, the H69D mutation in the context of the M6 precursor behaved the same as the D25N mutation (lanes 28, 29). Collectively, the L-MBP appeared to influence H69D precursor autoprocessing in a p6*-independent manner.

### MBP SigP is sufficient to modulate precursor autoprocessing

**Fig 3:**
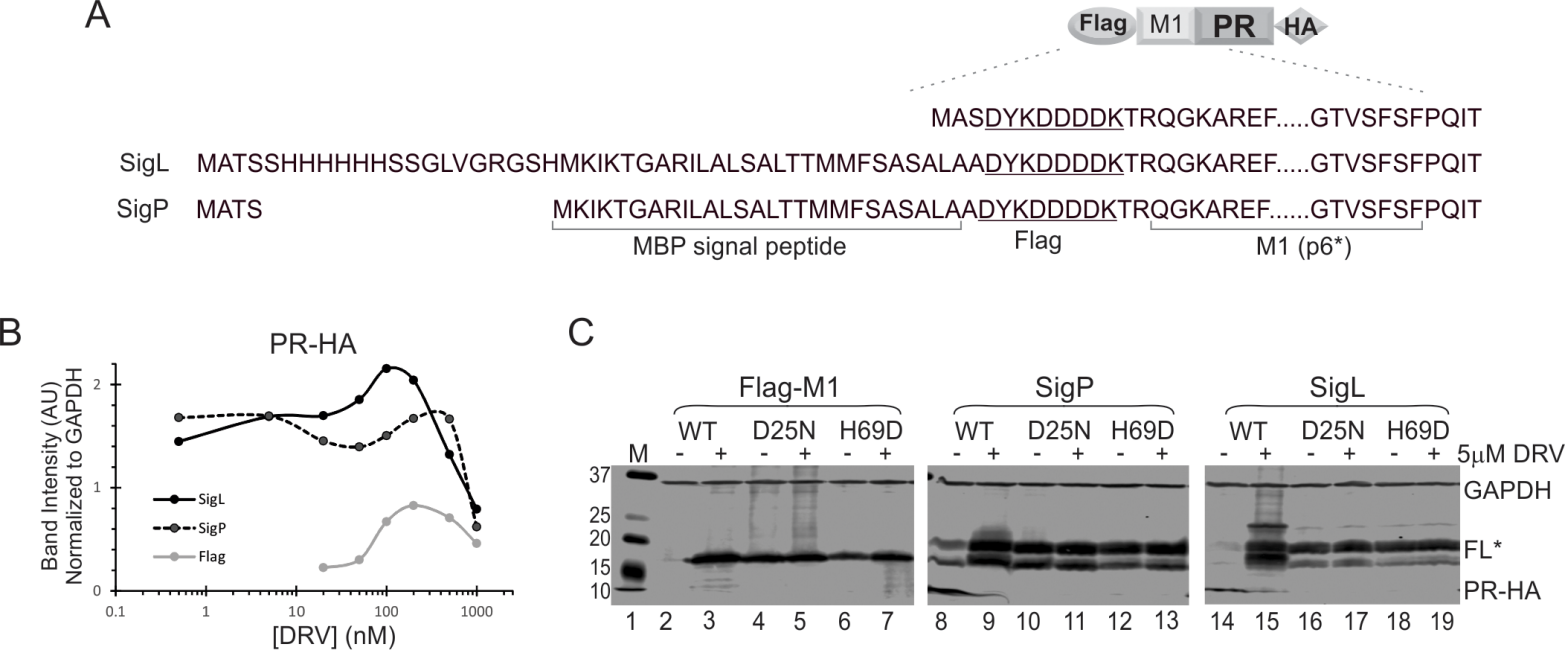
The MBP SigP is sufficient at modulating precursor autoprocessing. **(A)** Schematic diagram of the tested mini-fusion precursors. Flag-M1-PR-HA is the parental construct to which various signal sequences were added N-terminally. (**B)** Quantitative comparison of the mature PRs released from the indicated precursors. The graph is representative of five independent experiments. (**C)** Influences of SigL and SigP on H69D autoprocessing activity.

An obvious difference between L-MBP (or F-MBP) and GST (or C2-MBP) is the MBP SigP (Fig 1B). To determine whether the SigP by itself is sufficient to alter the autoprocessing outcome, we first constructed and tested Flag-M1-PR-HA that does not have any bulky fusion tag at the N-terminus and has the distal cleavage site truncated (Fig 3A). This precursor autoprocessed effectively like the GST fusion precursor (S1 Fig) and was used as the parent precursor in the subsequent experiments. The mature PR released from the Flag-M1 precursor displayed a typical bell-shaped detection profile similar to that made by GST precursors (Fig 3B, gray line; S2 Fig). When treated with micromolar indinavir, these two constructs also released additional HA-reactive bands that ran to the positions between mature PR and an intermediate designated as p6*-PR^***b***^ in a previous report [25] and below. We did not further characterize these bands as they were only detectable at high concentrations of protease inhibitors. Compared to Flag-M1-PR-HA, SigL and SigP have N-terminal extensions both containing the MBP signal peptide (Fig 3A). The mature PRs released from SigL and SigP precursors were easily detectable in the absence of PR inhibitor and exhibited detection profiles like those released from L-MBP or F-MBP fusion precursors in response to DRV treatment (Fig 3B; S2 Fig). Our data demonstrated that inclusion of the MBP SigP was sufficient to alter the self-degradation property of the released mature PRs.

We also examined several H69D-containing precursors as another readout of autoprocessing modulation. The WT Flag-M1 was autoprocessing competent and thus showed little or no detectable full-length precursor unless treated with 5μM DRV to suppress autoprocessing (Fig 3C lanes 2, 3). The D25N protease-deficient Flag-M1 precursor was autoprocessing deficient showing approximately equal amounts of unprocessed precursor with or without 5μM DRV (Fig 3C lanes 4, 5). The Flag-M1 precursor bearing the H69D mutation reproducibly exhibited partial activity (Fig 3C lanes 6, 7) as about 60% full-length precursor was detected without 5μM DRV compared to with 5μM DRV treatment. In contrast, with SigP or SigL fusion, H69D was mostly autoprocessing-deficient, similar to the D25N controls, showing no difference in detection of the full-length precursor with or without 5 μM DRV (Fig 3C lanes 12, 13, 18, 19). Therefore, the MBP SigP alone was sufficient to abolish H69D autoprocessing.

The MBP SigP consists of an N-terminal basic hydrophilic segment (SB) followed by a hydrophobic core (SHC). The last six residues are believed to be recognized by cellular signal peptidases [26]. To determine whether there is a specific linear motif responsible for the observed modulation, we engineered mini fusion precursors carrying different segments (SB or SHC) of MBP SigP (S3 Fig, panel A). The SR2a sequence was previously reported to retain all the essential features required for MBP export in *E*. *coli* despite missing a portion of the hydrophobic core; the SR2b sequence, carrying a point mutation compared to the SR2a, is deficient in MBP export [27]. Both SHC and SR2a precursors maintained the ability to produce mature PRs resistant to self-degradation (S3 Fig, panel B, lanes 7–10). In contrast, the mature PRs made by SB and SR2b were mostly degraded in the absence of PR inhibitor and only became detectable following 0.2μM DRV treatment (S3 Fig, panel B, lanes 5, 6, 11, 12). Therefore, SHC and SR2a retained the modulatory effect of releasing self-degradation-resistant PRs whereas SB and SR2b did not.

In the context of the SHC and SR2a fusions, H69D autoprocessing was mostly abolished (S3 Fig, lanes 27–30). These two constructs also showed trace amounts of mature PR, but quantification analysis showed no difference in full-length precursor detection with or without 5μM DRV treatment, indicating that these H69D precursors were as inactive as they were when treated with 5 μM DRV. In the context of SB, H69D autoprocessing was partially active (S3 Fig, lane 25), which was like the Flag-M1 H69D control (Fig 3C, lane 6). In the context of SR2b, H69D demonstrated a phenotype like SR2a in that similar amounts of the FL precursor were detected with or without 5μM DRV. Collectively, our data demonstrated that the hydrophobic core (SHC) alone could induce the observed modulation effects but the N-terminal basic hydrophilic segment (SB) by itself was not. Furthermore, SR2a could recapitulate SHC’s effects whereas SR2b displayed a mixed phenotype as it released self-degradation-sensitive mature PR (S3 Fig, lanes 11, 12) but suppressed H69D autoprocessing (S3 Fig, lanes 31, 32). Note that SR2a and SR2b differ by only one residue at position 8 (R8L in SR2a and R8P in SR2b) and SR2a is competent in MBP export but SR2b is not [27]. Collectively, these results support the notion that precursor autoprocessing can be modulated by functional determinants like SHC or SR2a. However, no new linear motif was identified.

### Autoprocessing modulation influenced by SigP position

**Fig 4:**
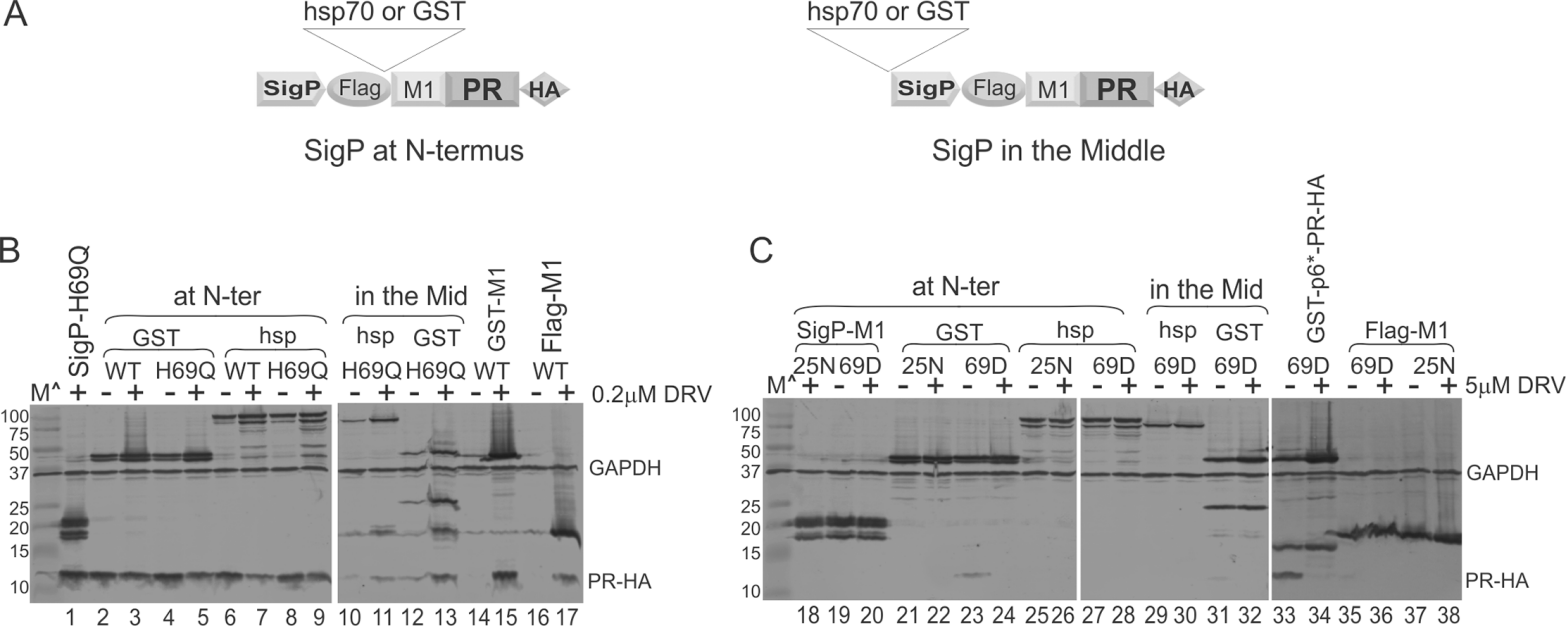
Position dependence of MBP SigP on modulation of autoprocessing. **(A)** Schematic diagram of GST- or hsp70- fusion precursors with MBP SigP placed at the very N-terminus (*left*) or in the middle between the fusion tag and M1-PR (*right*). (**B, C)** Influences of MBP SigP position on self-degradation of released mature PR (*panel B*) and on H69D autoprocessing activity (*panel C*).

Within L-MBP and F-MBP fusion precursors, the MBP SigP is separated from the p6*-PR by C2-MBP protein (~38 kD), suggesting that its effect is long range at the primary sequence level. We sought to determine whether the MBP SigP could also exert the same modulation effects on PR when an unrelated protein was inserted between them. GST (~25 kD) and hsp70 (~ 70 kD) were chosen for this test as they were previously utilized as fusion tags [8]. Our data demonstrated that the MBP SigP at the N-terminus retained its ability to modulate autoprocessing outcomes even when it was separated by GST or hsp70 from M1-PR. The released mature PRs were self-degradation resistant (Fig 4B, lanes 3–9) and H69D mutation abolished precursor autoprocessing as effectively as a D25N mutation (Fig 4C, lanes 21–28). The mature PRs released from the control GST or hsp70 fusion precursor without the MBP SigP were rapidly self-degraded in the absence of protease inhibitor and were only detectable when treated with 0.2μM DRV (Fig 4B, lanes 14–17). Also, the H69D fusion precursors without MBP SigP showed partial autoprocessing activities (lanes 33–36). Therefore, the p6*-PR mediated autoprocessing can be modulated by the MBP SigP even when hsp70 was inserted in between.

We then determined if MBP SigP would maintain the modulatory effects when placed in the middle of a fusion precursor immediately upstream of p6*-PR. Surprisingly, although the signal was closer to p6*-PR in the linear sequence, it failed to induce all the previously observed effects. The released mature PRs were susceptible to self-degradation unless suppressed with 0.2μM DRV (lanes 10–13), like the control samples without the MBP SigP (lanes 14–17). On the other hand, the inhibitory effect on H69D autoprocessing was maintained as similar levels of full length precursors were detected with or without 5μM DRV (lanes 29–32). This observation suggested that production of self-degradation resistant mature PR is not necessarily coupled with complete suppression of H69D autoprocessing. Collectively, the results confirmed that the MBP SigP can impact precursor autoprocessing over a long distance to modulate the self-degradation property of the released mature PR when placed at the N-terminus. The MBP SigP can also impede the H69D autoprocessing activity in a position-independent manner.

### N-terminal SigP alters precursor distribution

**Fig 5:**
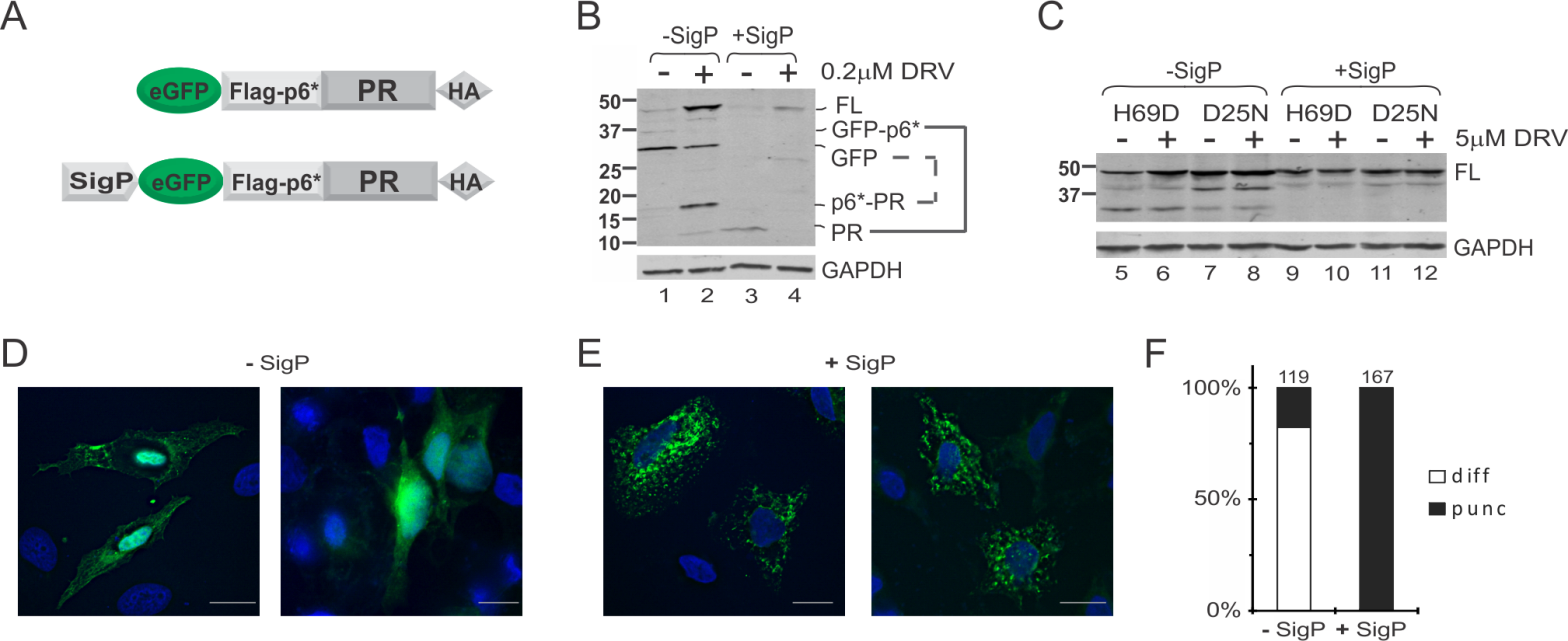
MBP SigP alters subcellular distribution of GFP fusion precursors. **(A)** Schematic diagram of GFP fusion precursors with or without MBP SigP at the N-terminus. (**B, C)** Western blot assessment of MBP SigP’s influences on mature PR self-degradation (*panel B*) and H69D autoprocessing (*panel C*) in transfected HeLa cells. (**D, E)** Representative images of HeLa cells transfected with plasmids encoding for the D25N GFP fusion without (*panel D*) or with (*panel E*) MBP SigP. Bars: 1 μm. (**F)** Quantitative analysis of different GFP staining patterns in transfected HeLa cells. The numbers above the bars denote the total GFP positive cells analyzed.

To gain further insights into the mechanism underlying the modulation exerted by MBP SigP, we examined the subcellular distribution of fusion precursors tagged with GFP with or without the MBP SigP (Fig 5A). Autoprocessing properties of these GFP fusion precursors in transfected HeLa resembled to those observed in transfected HEK293T (Fig 5B and 5C). The mature PR released from the GFP-tagged precursor without MBP SigP was not detectable in the absence of protease inhibitor; it became detectable when treated with 0.2μM DRV (Fig 5B, lanes 1, 2). In contrast, the mature PR made by the GFP-tagged precursor with SigP was readily detectable in the absence of protease inhibitor (Fig 5B, lane 3). Without SigP, H69D mutation partially suppressed precursor autoprocessing (Fig 5C, lanes 5, 6); with SigP, H69D abolished precursor autoprocessing as effectively as D25N (Fig 5C, lanes 9–12).

Having confirmed that the GFP tag did not alter precursor autoprocessing ability, GFP fusions carrying D25N (to prevent autoprocessing) with or without SigP were examined in transfected HeLa cells by confocal microscopy. Interestingly, the two precursors exhibited distinct distribution patterns. Without SigP, distribution was mostly diffused; a small percentage of the cells exhibited small puncta (Fig 5D). In contrast, precursors with SigP displayed bright clusters that appeared to surround vesicle-like structures (Fig 5E). We interpreted this to suggest that SigP mediates precursor targeting to certain membrane vesicles where precursor autoprocessing is indirectly modulated by cellular factors associated with the local environment. The fact that the GFP-tagged fusion precursors were readily detectable excludes their association with organelles such as the lysosome where GFP signals would be quenched by the low pH environment of the lumen. Rather, the results suggest that the subcellular environment modulates precursor autoprocessing.

### Context-dependent autoprocessing of precursors with mutations identified in variants from HIV-1-infected individuals

**Fig 6:**
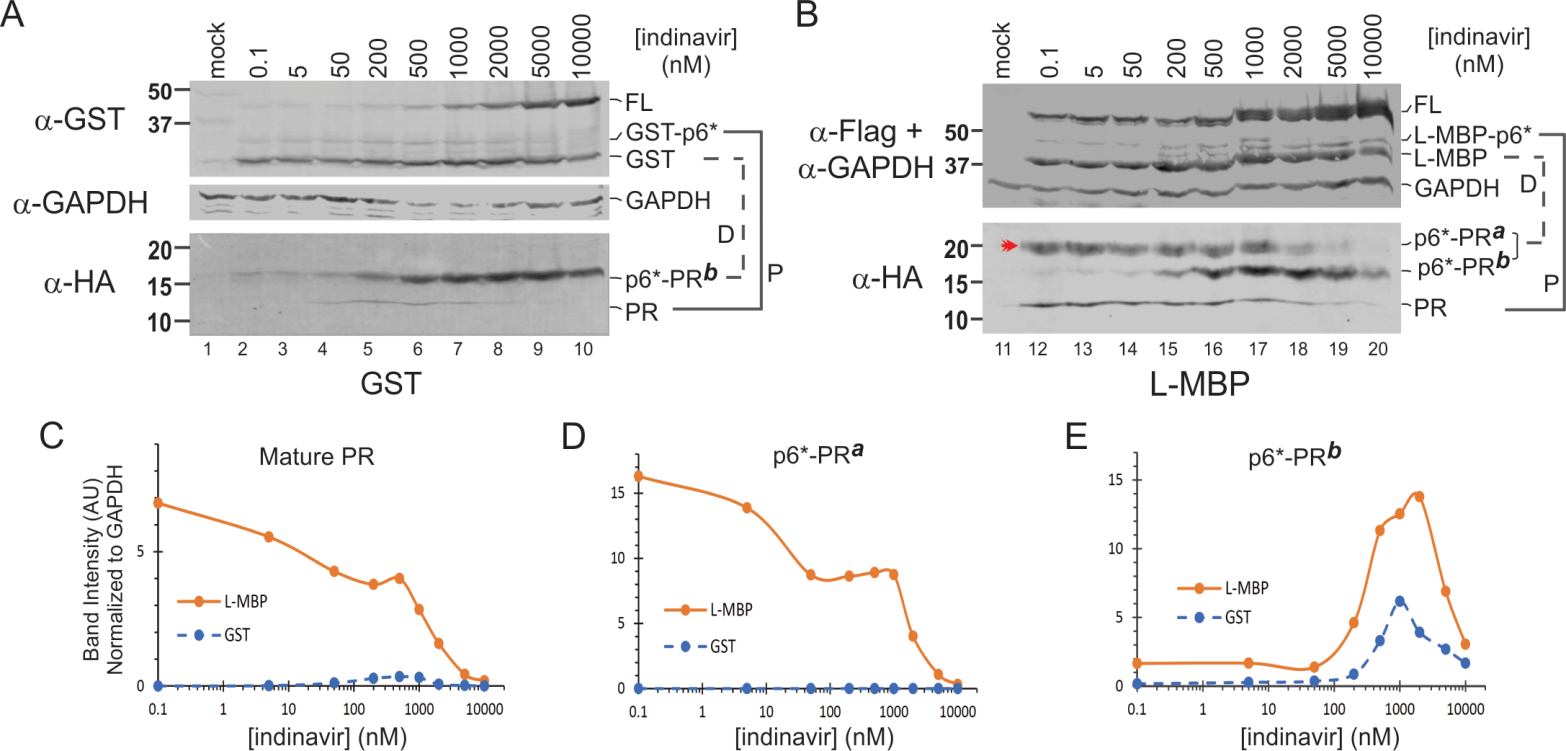
**Autoprocessing of F56C precursors in the context of GST (A) and L-MBP (B) fusion.** Lysates collected from transfected HEK293T cells were resolved by SDS-PAGE and probed with the indicated antibodies for visualization of full-length (FL) precursors and autoprocessing products. The corresponding proximal (P) and distal (D) processing products are denoted and connected with solid or dashed lines, respectively. The double arrow head (panel B, HA blot) indicates a distal processing product unique to the context of L-MBP fusion. Band intensity of mature PR (C), p6*-PR^**a**^ (D), and p6*-PR^**b**^ (E) normalized to GAPDH was plotted to IDV concentrations, respectively.

We recently described point mutations that emerged in the HIV-1 viruses isolated from a subpopulation of Women’s Interagency HIV Study (WIHS) participants where anti-retroviral agents, including indinavir (IDV), had failed to suppress the viral load [28, 29]. One of these is a substitution of the C-terminal p6* residue phenylalanine 56 (F56) for cysteine (C; F56C), which thereby alters the P1 residue at the PR N-terminal cleavage site [25]. We constructed precursors carrying this point mutation with GST *vs*. L-MBP fusion tags to further test if context-dependent autoprocessing is a general property (Fig 6). Our results showed that the mature PR released from the GST fusion precursor displayed a typical bell-shaped detection profile in response to IDV treatment whereas the mature PR released from L-MBP fusion precursor was readily detectable in the absence of any PI and its detection remained steady up to 1μM IDV (Fig 6C). This was similar to the wild type precursors (*c*.*f*., Fig 1C), confirming that the mature PRs released from GST vs L-MBP precursors differed in their self-degradation propensity.

Distal processing of this mutation was also differentially affected by these two tags. The GST precursor produced one distal processing product p6*-PR^**b**^ (Fig 6A, lower panel) while the L-MBP precursor released two (Fig 6B, lower panel). N-terminal sequencing analysis confirmed that both p6*-PR^**a**^ and p6*-PR^**b**^ had the same N-terminal sequence (PQGKA) despite their different mobility in SDS-PAGE. Of note, the p6*-PR^**a**^ fragment was specific to L-MBP fusion (Fig 6D) and appeared to be stable over a wide range of IDV concentrations (up to 2μM). The p6*-PR^b^ product displayed a bell-shaped detection profile in response to IDV treatment (Fig 6E) as seen with the wild type precursor [8]. Consequently, these results support the idea that precursor autoprocessing is differentially modulated by GST vs L-MBP tags leading to liberation of products with distinct detection profiles.

### Autoprocessing products from the NL4-3 viral particles resemble those released from the L-MBP fusion precursors

**Fig 7:**
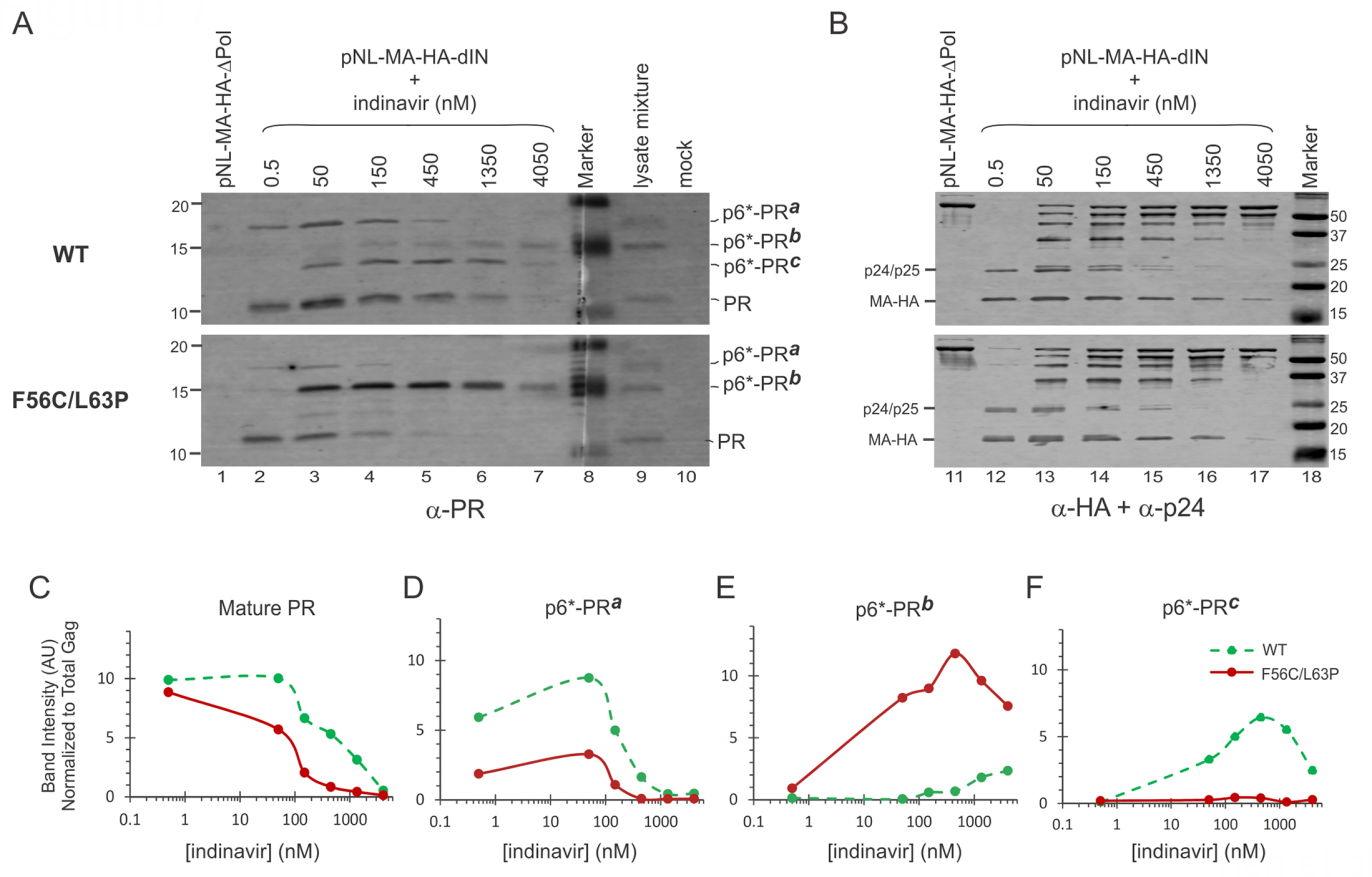
Detection and quantification of PRs in viral particles made by the WT and F56C/L63P proviral constructs. **(A)** The viral particles were probed with a polyclonal PR antibody. A mixture of lysates collected from cells transfected with L-MBP precursors without any C-terminal fusion epitope was included to serve as size references of mature PR, p6*-PR^**a**^, and p6*-PR^**b**^ fragment (lane 9). (**B)** The same viral particle samples at 5-fold less amounts were resolved in parallel, probed with a HA-antibody first followed by p24 probing. (**C-F)** quantification of PR-reactive bands normalized to total HA signals representing the total Gag.

In the light of our results showing that precursor autoprocessing is context-dependent, we sought to examine autoprocessing products associated with viral particles released from 293T cells transfected with a NL4-3 derived proviral construct (pNL-MA-HA-ΔIN) (Fig 7). The wild type control (Fig 7A and 7B, the upper panels) was compared to a F56C/L63P double mutation in the proviral context. This double mutation displayed phenotypes resembling the F56C single mutation that supported normal Gag processing even with reduced mature PR production due to the suboptimal cleavage site rendered by the F56C mutation at the P1 position [25]. The viral particle-associated mature PR and other PR-containing fragments, likely p6*-PR variants, were probed with a rabbit anti-PR antibody (Fig 7A). In parallel, the same samples were probed with an anti-HA antibody followed by anti-p24 antibody probing to determine the total Gag proteins and Gag processing (Fig 7B). Our results showed that the mature PR in the WT particles was readily detectable and remained so at low IDV concentrations, resembling the mature PR released by the L-MBP fusion precursor. The F56C/L63P double mutation produced less mature PR than the WT control (Fig 7C). Using the p6*-PR^a^ and p6*-PR^**b**^ made by the L-MBP fusion precursor as size references (Fig 7A, lane 9), we found several p6*-PR fragments displaying various response profiles to IDV treatment. The p6*-PR^**a**^ fragment was detected in both constructs with the WT releasing more than the double mutation (Fig 7D). Meanwhile, the F56C/L63P produced more p6*-PR^**b**^ fragment than the WT (Fig 7E) but had much less p6*-PR^**c**^ than the WT (Fig 7F). Collectively, these data demonstrated the existence of mature PR along with several p6*-PR fragments in viral particles produced in the context of a proviral construct. The SigP-containing precursors therefore appear to recapitulate an autoprocessing property that closely resembles that of the virus.

## Discussion

### Mature PRs with diverse self-degradation propensities

Autoproteolysis (self-degradation) is often observed with *in vitro* purified recombinant mature PRs [6, 10, 11, 19, 20] and thus it is widely accepted that all mature PRs are prone to rapid self-degradation. In this report, mature PRs with the same amino acid sequence but distinct (sensitive *vs* resistant) self-degradation properties were detected from precursors with different fusion tags (GST *vs* L-MBP). Accordingly, we suggest that precursor autoprocessing can undergo more than one pathway induced by different contexts leading to production of mature PRs with diverse self-degradation propensities. Of an interesting note, the NL4-3 associated mature PRs were also resistant to self-degradation as the particles were collected 48h post transfection and subjected to a 2h centrifugation step prior to lysis of the particles with SDS in preparation for Western analysis (Fig 7A). In this regard, the L-MBP fusion (or the MBP SigP at the N-terminus) is a better mimic of the viral context than the GST or other fusions in regard to liberation of mature PRs that resist self-degradation.

### MBP SigP modulation mechanism

The MBP SigP is known to mediate MBP translocation across the plasma membrane into the periplasmic space in *E coli*. However, its biology in mammalian cells is unclear in terms of whether it would mediate targeting of specific membrane organelles and the subsequent trans-membrane translocation. We speculated that MBP SigP-mediated translation initiation and/or membrane targeting might contribute to the different autoprocessing outcomes between precursors with or without it. Once the SigP is synthesized and emerges from the ribosome, it might interact with mammalian proteins that influence its subcellular distribution and/or folding state. When placed in the middle, the SigP accessibility to these cellular components would be reduced. Consistent with this speculation, our subcellular distribution analysis revealed that MBP SigP targeted the GFP fusion precursor to certain vesicle-like structures, probably at the cytoplasmic site (outer rims); whereas the precursor without SigP was diffused throughout the cytoplasm (Fig 7). Given that Gag/Gag-Pol assembly happens at the cytoplasmic side of the plasma membrane, Gag-Pol precursor autoprocessing could be subjected to modulation by cellular/viral factors enriched/specific to the assembly sites as well. Collectively, it appears that different autoprocessing outcomes might be correlated with different subcellular locations where they could vary in molecule composition, pH, redox status etc, revealing that precursor autoprocessing is context-dependent and responsive to different subcellular environments.

### Context-dependent production of p6*-PR fragments

Many fusion precursors tested in this study carry both distal (D) and proximal (P) cleavage sites (Fig 1). Proximal site processing releases mature PR and distal site processing produces p6*-PR. This report demonstrated that various p6*-PR fragments were detected in different contexts: the GST fusion resulted in production of mainly p6*-PR^**b**^ fragment (Fig 6A); the L-MBP fusion led to production of both p6*-PR^**a**^ and p6*-PR^**b**^ fragments (Fig 6B) and the NL4-3 particles contained several p6*-PR fragments (Fig 7A). Furthermore, the p6*-PR fragments released from different sequences (*e*.*g*., wt *vs* F56C/L63P) also exhibited diverse IDV response profiles (Fig 7D–7F), indicating that precursor autoprocessing could be influenced by different PR sequences leading to liberation of various products We and others previously reported that a p6*-PR fragment resulting from a mutated proximal site can partially process Gag polyprotein [4], suggesting that these p6*-PR fragments could contribute to Gag processing during virion maturation. In this regard, it is interesting to note that F56C/L63P generated more p6*-PR^**b**^ (Fig 7E) but less mature PR, p6*-PR^**a**^, and p6*-PR^**c**^ than the WT control. The fact that this double mutation displayed normal Gag processing and viral infectivity [29] suggests a tag team strategy by which these p6*-PRs and the mature PR work together cooperatively to make infectious virions. This strategy could also contribute to development of drug resistance as these p6*-PR fragments were detected at high IDV concentrations and their production was less sensitive to inhibition by PR inhibitors [7, 8, 13].

In summary, this report demonstrated that precursor autoprocessing activity and its outcomes are context-dependent. We speculate that the Gag-Pol precursor is structurally and enzymatically flexible such that it can take on different autoprocessing pathways under different contexts leading to diverse outcomes. In line with this concept, Zybarth et al previously observed that a partial truncation of the nucleocapsid (NC) domain of a forced-frameshift Gag-PR precursor interfered with precursor autoprocessing but deletion of both NC and most p6* rescued and enhanced precursor autoprocessing (data not shown). As another example, a recombinant MBP-fused precursor expressed in *E*. *coli* was autoprocessing-deficient but became autoprocessing-competent following an *in vitro* denaturation/renaturation cycle [30]. In this case, different cellular contexts (mammalian vs prokaryote) led to different autoprocessing outcomes, supporting the hypothesis that precursor autoprocessing is regulated by sequences outside of the p6*-PR region *in cis* and/or by other cellular factors *in trans*. Additionally, Yu et al reported that enhanced Gag-Pol autoprocessing induced by replacement of the p6* domain with a leucine zipper was suppressed by the N-terminal tetra-peptide [31]. In addition to revealing the complexity of autoprocessing regulation, our findings underscore the necessity of employing physiologically relevant contexts to study the autoprocessing mechanism.

## Supporting information

S1 Fig Autoprocessing of precursors with or without GST at the N-terminus. **A:** Schematic diagram of the fusion precursors. **B:** Transfected HEK 293T cells were treated with increasing concentrations of DRV (upper panels) or SQV (lower panels) for 24h. Cell lysates were examined with mouse anti-Flag and anti-HA antibodies. The solid and dotted lines connect the products released from proximal (P) and distal (D) processing, respectively. The circle in panel B denotes indicates a nonspecific band that co-migrated with the GST-Flag-p6* product.
(TIF)

S2 Fig DRV sensitivity of mini fusion precursors with or without SigP. Transfected HEK 293T cells were treated with DRV at the indicated concentrations for 24h. Cell lysates were analyzed by SDS-PAGE followed by western blotting with mouse anti-HA and anti-GAPDH antibodies. The asterisks indicate the full-length precursors.
(TIF)

S3 Fig Effects of SigP fragments on precursor autoprocessing. **A:** Schematic diagram of the tested mini-fusion precursors. Flag-M1-PR-HA is the parental construct to which various signal sequences were added N-terminally. **B, C:** Influenced of SigP fragments on mature PR with or without 0.2μM DRV to suppress mature PR self-degradation (*panel B*), and on H69D autoprocessing with or without 5μM DRV to block precursor autoprocessing (*panel C*).
(TIF)

S1 File Coding sequences of the listed plasmids used in this study. The sequences are from the start to stop codon.
(DOCX)
