# Supplementary figures and images for: Context-dependent autoprocessing of human immunodeficiency virus type 1 protease precursors
Source: PLoS One. 2018 Jan 16;13(1):e0191372. doi: 10.1371/journal.pone.0191372 (PMC5770051; doi:10.1371/journal.pone.0191372)

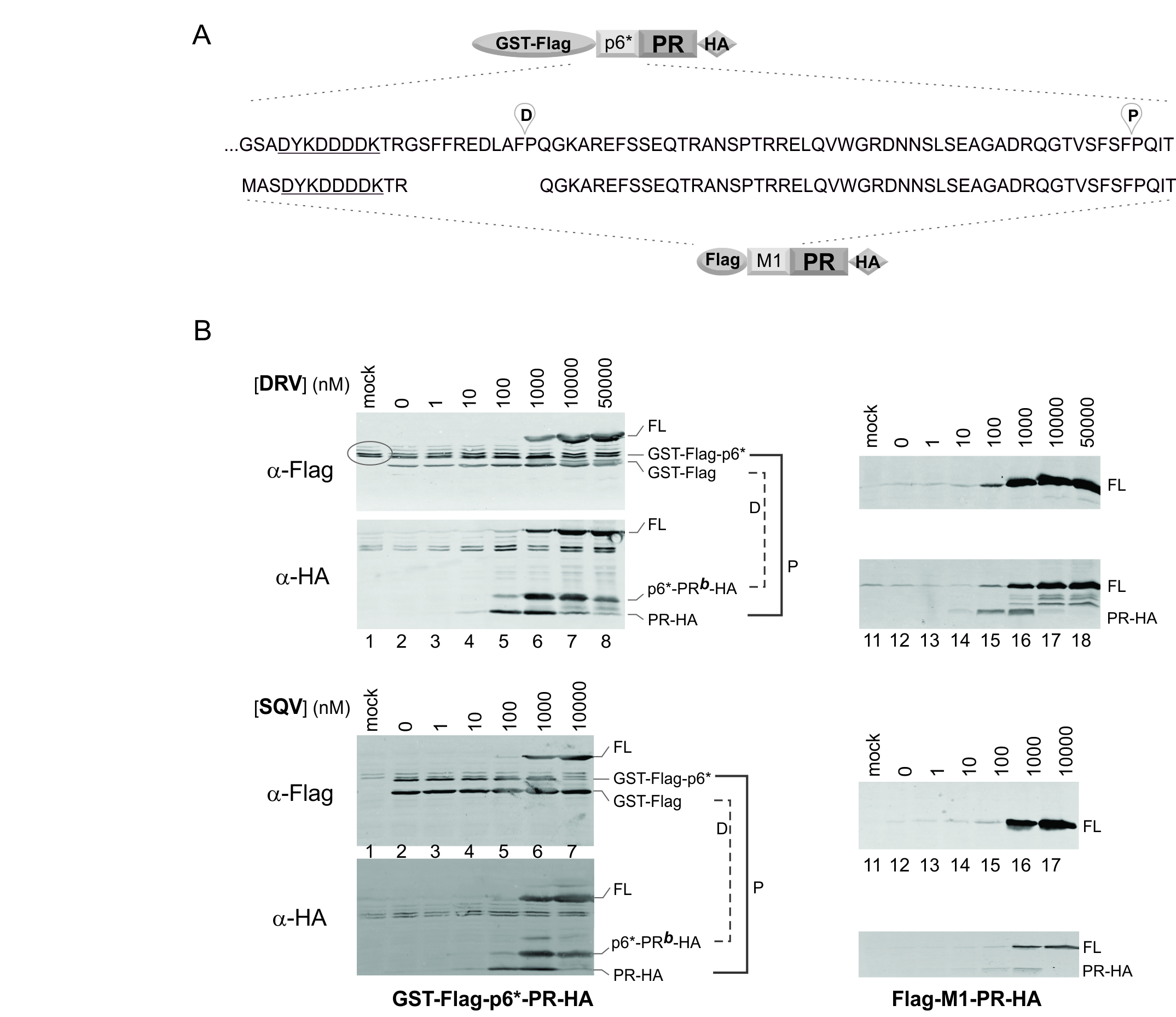

Supplement: S1 Fig — A: Schematic diagram of the fusion precursors. B: Transfected HEK 293T cells were treated with increasing concentrations of DRV (upper panels) or SQV (lower panels) for 24h. Cell lysates were examined with mouse anti-Flag and anti-HA antibodies. The solid and dotted lines connect the products released from proximal (P) and distal (D) processing, respectively. The circle in panel B denotes indicates a nonspecific band that co-migrated with the GST-Flag-p6* product. (TIF) [file pone.0191372.s001.tif]

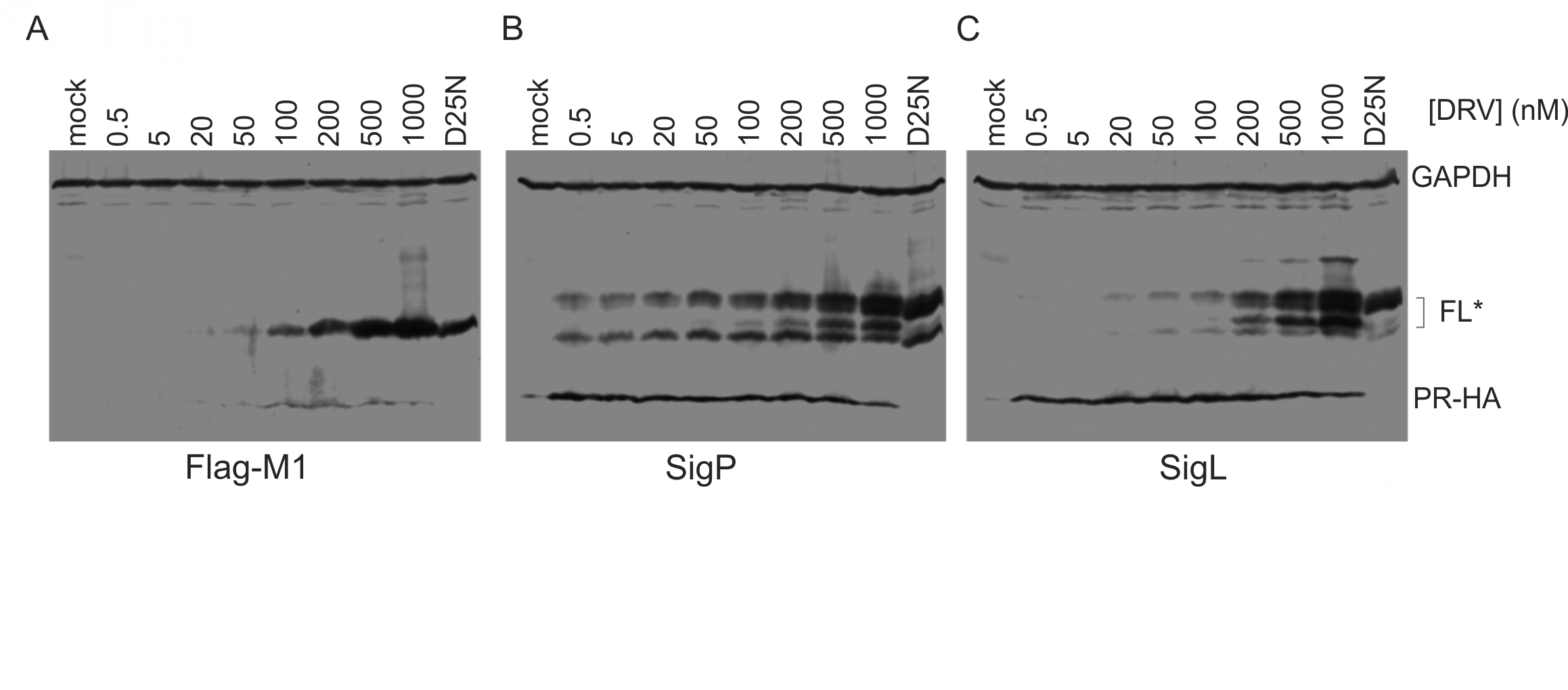

Supplement: S2 Fig — Transfected HEK 293T cells were treated with DRV at the indicated concentrations for 24h. Cell lysates were analyzed by SDS-PAGE followed by western blotting with mouse anti-HA and anti-GAPDH antibodies. The asterisks indicate the full-length precursors. (TIF) [file pone.0191372.s002.tif]

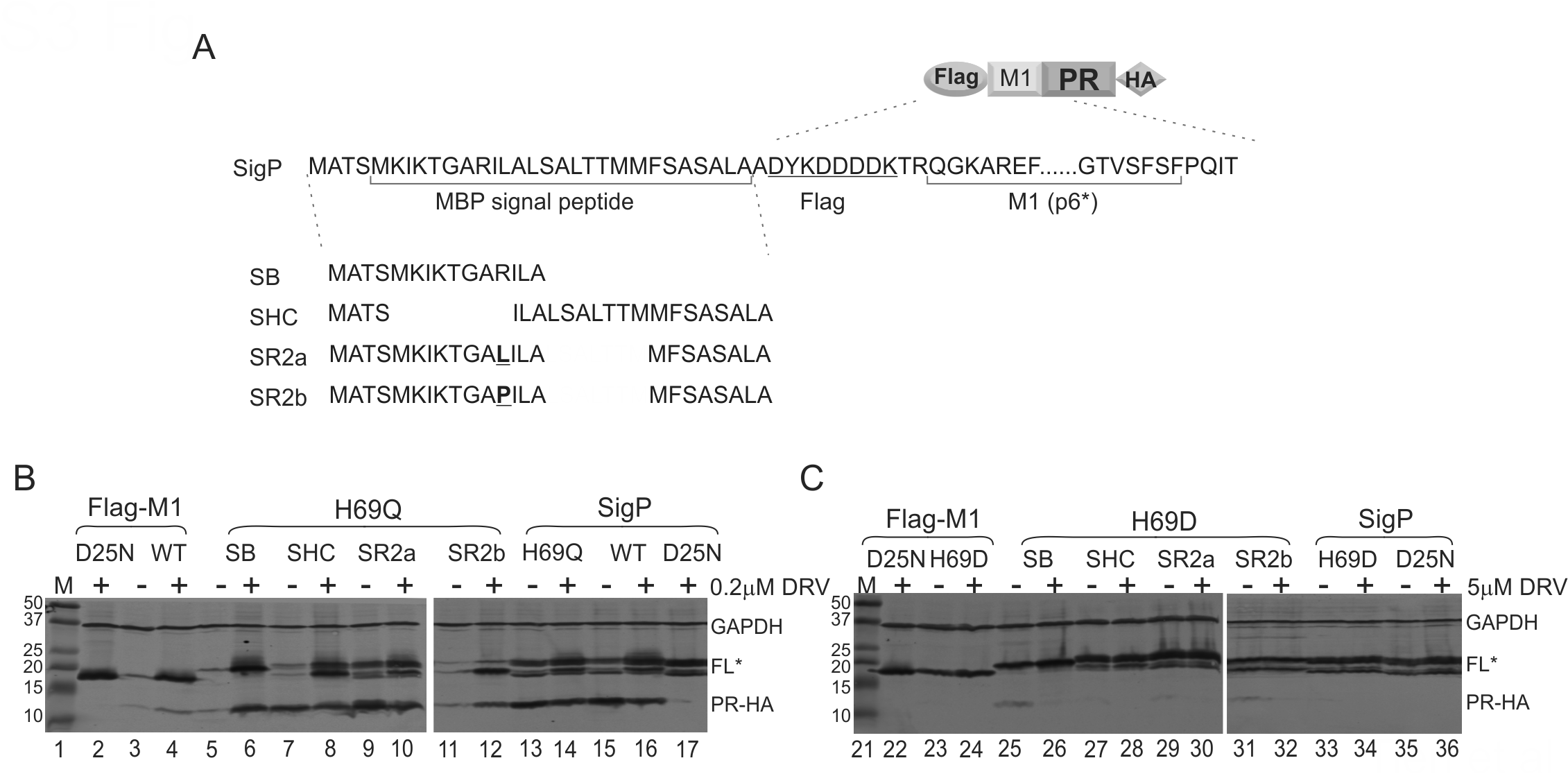

Supplement: S3 Fig — A: Schematic diagram of the tested mini-fusion precursors. Flag-M1-PR-HA is the parental construct to which various signal sequences were added N-terminally. B, C: Influenced of SigP fragments on mature PR with or without 0.2μM DRV to suppress mature PR self-degradation (panel B), and on H69D autoprocessing with or without 5μM DRV to block precursor autoprocessing (panel C). (TIF) [file pone.0191372.s003.tif]
