# Supplementary material for: Context-dependent autoprocessing of human immunodeficiency virus type 1 protease precursors
Source: PLoS One. 2018 Jan 16;13(1):e0191372. doi: 10.1371/journal.pone.0191372 (PMC5770051; doi:10.1371/journal.pone.0191372)
Supplement: S1 File — The sequences are from the start to stop codon. (DOCX) [file pone.0191372.s004.docx]

The coding sequences of the listed plasmids

**F-MBP-Flag-M5-PR-HA:**

atggccactagtatgaaaataaaaacaggtgcacgcatcctcgcattatccgcattaacgacgatgatgttttccgcctcggctctcgccaaaatcgaagaaggtaaactggtaatctggattaacggcgataaaggctataacggtctcgctgaagtcggtaagaaattcgagaaagataccggaattaaagtcaccgttgagcatccggataaactggaagagaaattcccacaggttgcggcaactggcgatggccctgacattatcttctgggcacacgaccgctttggtggctacgctcaatctggcctgttggctgaaatcaccccggacaaagcgttccaggacaagctgtatccgtttacctgggatgccgtacgttacaacggcaagctgattgcttacccgatcgctgttgaagcgttatcgctgatttataacaaagatctgctgccgaacccgccaaaaacctgggaagagatcccggcgctggataaagaactgaaagcgaaaggtaagagcgcgctgatgttcaacctgcaagaaccgtacttcacctggccgctgattgctgctgacgggggttatgcgttcaagtatgaaaacggcaagtacgacattaaagacgtgggcgtggataacgctggcgcgaaagcgggtctgaccttcctggttgacctgattaaaaacaaacacatgaatgcagacaccgattactccatcgcagaagctgcctttaataaaggcgaaacagcgatgaccatcaacggcccgtgggcatggtccaacatcgacaccagcaaagtgaattatggtgtaacggtactgccgaccttcaagggtcaaccatccaaaccgttcgttggcgtgctgagcgcaggtattaacgccgccagtccgaacaaagagctggcaaaagagttcctcgaaaactatctgctgactgatgaaggtctggaagcggttaataaagacaaaccgctgggtgccgtagcgctgaagtcttacgaggaagagttggcgaaagatccacgtattgccgccactatggaaaacgcccagaaaggtgaaatcatgccgaacatcccgcagatgtccgctttctggtatgccgtgcgtactgcggtgatcaacgccgccagcggtcgtcagactgtcgatgaagccctgaaagacgcgcagactgctagccaaggaactgtatcctttagcttccctcagatcactctttggcagcgacccctcgtcacaataaagataggggggcaattaaaggaagctctattagatacaggagcagatgatacagtattagaagaaatgaatttgccaggaagatggaaaccaaaaatgatagggggaattggaggttttatcaaagtaggacagtatgatcagatactcatagaaatctgcggacataaagctataggtacagtattagtaggacctacacctgtcaacataattggaagaaatctgttgactcagattggctgcactttaaattttgtcgactacccatatgacgtcccggactactaa

**C2-MBP-Flag-p6*-PR-HA:**

atggccactagtatgaaaatcgaagaaggtaaactggtaatctggattaacggcgataaaggctataacggtctcgctgaagtcggtaagaaattcgagaaagataccggaattaaagtcaccgttgagcatccggataaactggaagagaaattcccacaggttgcggcaactggcgatggccctgacattatcttctgggcacacgaccgctttggtggctacgctcaatctggcctgttggctgaaatcaccccggacaaagcgttccaggacaagctgtatccgtttacctgggatgccgtacgttacaacggcaagctgattgcttacccgatcgctgttgaagcgttatcgctgatttataacaaagatctgctgccgaacccgccaaaaacctgggaagagatcccggcgctggataaagaactgaaagcgaaaggtaagagcgcgctgatgttcaacctgcaagaaccgtacttcacctggccgctgattgctgctgacgggggttatgcgttcaagtatgaaaacggcaagtacgacattaaagacgtgggcgtggataacgctggcgcgaaagcgggtctgaccttcctggttgacctgattaaaaacaaacacatgaatgcagacaccgattactccatcgcagaagctgcctttaataaaggcgaaacagcgatgaccatcaacggcccgtgggcatggtccaacatcgacaccagcaaagtgaattatggtgtaacggtactgccgaccttcaagggtcaaccatccaaaccgttcgttggcgtgctgagcgcaggtattaacgccgccagtccgaacaaagagctggcaaaagagttcctcgaaaactatctgctgactgatgaaggtctggaagcggttaataaagacaaaccgctgggtgccgtagcgctgaagtcttacgaggaagagttggcgaaagatccacgtattgccgccactatggaaaacgcccagaaaggtgaaatcatgccgaacatcccgcagatgtccgctttctggtatgccgtgcgtactgcggtgatcaacgccgccagcggtcgtcagactgtcgatgaagccctgaaagacgcgcagactgctagcgactacaaggacgatgacgacaagacgcgtggatccttttttagggaagatctggccttcccacaagggaaggccagggaattttcttcagagcagaccagagccaacagccccaccagaagagagcttcaggtttggggaagagacaacaactccctctcagaagcaggagccgatagacaaggaactgtatcctttagcttccctcagatcactctttggcagcgacccctcgtcacaataaagataggggggcaattaaaggaagctctattagatacaggagcagatgatacagtattagaagaaatgaatttgccaggaagatggaaaccaaaaatgatagggggaattggaggttttatcaaagtaggacagtatgatcagatactcatagaaatctgcggacataaagctataggtacagtattagtaggacctacacctgtcaacataattggaagaaatctgttgactcagattggctgcactttaaattttgtcgactacccatatgacgtcccggactactag

**Flag-M1-PR-HA:**

atggctagcgactacaaggacgatgacgacaagacgcgtcaagggaaggccagggaattttcttcagagcagaccagagccaacagccccaccagaagagagcttcaggtttggggaagagacaacaactccctctcagaagcaggagccgatagacaaggaactgtatcctttagcttccctcagatcactctttggcagcgacccctcgtcacaataaagataggggggcaattaaaggaagctctattagatacaggagcagatgatacagtattagaagaaatgaatttgccaggaagatggaaaccaaaaatgatagggggaattggaggttttatcaaagtaagacagtatgatcagatactcatagaaatctgcggacataaagctataggtacagtattagtaggacctacacctgtcaacataattggaagaaatctgttgactcagattggctgcactttaaattttgtcgactacccatatgacgtcccggactactag

**SigP-eGFP-Flag-p6*-PR-HA:**

atggccactagtatgaaaataaaaacaggtgcacgcatcctcgcattatccgcattaacgacgatgatgttttccgcctcggctctcgcaggatccgtgagcaagggcgaggagctgttcaccggggtggtgcccatcctggtcgagctggacggcgacgtaaacggccacaagttcagcgtgtccggcgagggcgagggcgatgccacctacggcaagctgaccctgaagttcatctgcaccaccggcaagctgcccgtgccctggcccaccctcgtgaccaccctgacctacggcgtgcagtgcttcagccgctaccccgaccacatgaagcagcacgacttcttcaagtccgccatgcccgaaggctacgtccaggagcgcaccatcttcttcaaggacgacggcaactacaagacccgcgccgaggtgaagttcgagggcgacaccctggtgaaccgcatcgagctgaagggcatcgacttcaaggaggacggcaacatcctggggcacaagctggagtacaactacaacagccacaacgtctatatcatggccgacaagcagaagaacggcatcaaggtgaacttcaagatccgccacaacatcgaggacggcagcgtgcagctcgccgaccactaccagcagaacacccccatcggcgacggccccgtgctgctgcccgacaaccactacctgagcacccagtccgccctgagcaaagaccccaacgagaagcgcgatcacatggtcctgctggagttcgtgaccgccgccgggatcactctcggcatggacgagctgtacaaggctagcgactacaaggacgatgacgacaagacgcgtggatccttttttagggaagatctggccttcccacaagggaaggccagggaattttcttcagagcagaccagagccaacagccccaccagaagagagcttcaggtttggggaagagacaacaactccctctcagaagcaggagccgatagacaaggaactgtatcctttagcttccctcagatcactctttggcagcgacccctcgtcacaataaagataggggggcaattaaaggaagctctattagatacaggagcagatgatacagtattagaagaaatgaatttgccaggaagatggaaaccaaaaatgatagggggaattggaggttttatcaaagtaggacagtatgatcagatactcatagaaatctgcggacataaagctataggtacagtattagtaggacctacacctgtcaacataattggaagaaatctgttgactcagattggctgcactttaaattttgtcgactacccatatgacgtcccggactactag

**SigL-Flag-M1-PR-HA:**

atggccactagtagccatcatcatcatcatcacagcagcggcctggtgccgcgcggcagccatatgaaaataaaaacaggtgcacgcatcctcgcattatccgcattaacgacgatgatgttttccgcctcggctctcgcagctgactacaaggacgatgacgacaagacgcgtcaagggaaggccagggaattttcttcagagcagaccagagccaacagccccaccagaagagagcttcaggtttggggaagagacaacaactccctctcagaagcaggagccgatagacaaggaactgtatcctttagcttccctcagatcactctttggcagcgacccctcgtcacaataaagataggggggcaattaaaggaagctctattagatacaggagcagatgatacagtattagaagaaatgaatttgccaggaagatggaaaccaaaaatgatagggggaattggaggttttatcaaagtaagacagtatgatcagatactcatagaaatctgcggacataaagctataggtacagtattagtaggacctacacctgtcaacataattggaagaaatctgttgactcagattggctgcactttaaattttgtcgactacccatatgacgtcccggactactag

**SigP-SHC-M1-PR-HA**:

atggccactagtatcctagcattatccgcattaacgacgatgatgttttccgcctcggctctcgcagctgactacaaggacgatgacgacaagacgcgtcaagggaaggccagggaattttcttcagagcagaccagagccaacagccccaccagaagagagcttcaggtttggggaagagacaacaactccctctcagaagcaggagccgatagacaaggaactgtatcctttagcttccctcagatcactctttggcagcgacccctcgtcacaataaagataggggggcaattaaaggaagctctattagatacaggagcagatgatacagtattagaagaaatgaatttgccaggaagatggaaaccaaaaatgatagggggaattggaggttttatcaaagtaagacagtatgatcagatactcatagaaatctgcggacataaagctataggtacagtattagtaggacctacacctgtcaacataattggaagaaatctgttgactcagattggctgcactttaaattttgtcgactacccatatgacgtcccggactactag
